# The effect of valve noise on the quality of life of patients after mechanical mitral valve replacement in a Chinese population

**DOI:** 10.1186/s13019-019-0956-1

**Published:** 2019-07-19

**Authors:** Zhi-Nuan Hong, Jiang-Shan Huang, Li-Qin Huang, Hua Cao, Qiang Chen

**Affiliations:** 1 Department of Cardiovascular Surgery, Union Hospital, Fujian Medical University, Fuzhou, 350001 People’s Republic of China; 2 Department of Cardiac Surgery, Fujian Provincial Maternity and Children’s Hospital, Affiliated Hospital of Fujian Medical University, Fuzhou, 350001 People’s Republic of China; 3 Department of Public Health, Fujian Medical University, Fuzhou, 350001 People’s Republic of China

**Keywords:** Heart valve diseases, SF-36, Quality of life, Mitral valve replacement

## Abstract

**Objective:**

To investigate the effect of valve noise on the quality of life (QOL) in Chinese patients who underwent mechanical mitral valve replacement.

**Methods:**

We enrolled a total of 103 patients who underwent mechanical mitral valve replacement (MVR, CM valve in 52 patients, SJM valve in 51 patients) from January 2016 to December 2016 in our institution and used the SF-36 as an instrument to assess patients’ QOL.

**Results:**

Patients’ QOL improved over time. Patients who experienced disturbances due to valve noise had lower SF-36 scores in each scale, especially in general health, vitality, and mental health. Only 8.74% (*n* = 9) of patients complained of valve noise 1 year after the operation compared to 19.42% (*n* = 20) in the first month after the operation. The number of patients who experienced disturbances due to valve noise decreased over time, with a *P* value of 0.58. Logistic regression analysis showed that female patients those aged < 60 years old had a higher risk of experiencing disturbances due to valve noise. The valve type (CM vs SJM), body mass index (BMI) and valve size showed no significant differences in patients who experienced disturbances due to continuous valve noise. The SF-36 results were similar in the CM group and SJM group 1 year after the operation.

**Conclusions:**

QOL evaluated by the SF-36 improved over time in Chinese patients who underwent mechanical MVR. Age less than 60 years and female sex were high risk factors for experiencing disturbances due to valve noise. CM and SJM mechanical valves demonstrated similar valve noise levels and impact on QOL in patients who experienced mechanical MVR.

## Introduction

Approximately 18000 prosthetic heart valves are implanted, and half of them are of the mechanical type. Surgical outcomes and prosthetic heart valve improvements demonstrate that mechanical heart valves provide better hemodynamics, durability, antithrombogenicity and long-term survival than other types of heart valves [1, 2]. The quality of life (QOL) after valve replacement has also become an essential assessment of treatment effects [3, 4]. For a patient who undergoes mechanical valve replacement, the QOL may be affected by the following factors: the clicking sound of the mechanical valve, patients’ mental state, patients’ recognition of anticoagulation-related bleeding events and valve embolism. With the use of document retrieval, we found that few studies have been conducted to investigate the effect of valve noise on QOL after mitral mechanical valve replacement in Chinese populations. We aimed to use SF-36 as an instrument to assess patients’ QOL and to determine the risk factors affected by valve noise.

## Materials and methods

### Study design

In this study, we enrolled a total of 103 patients who underwent mechanical mitral valve replacement (CM valve in 52 patients, SJM valve in 51 patients) between January 2016 and December 2016. We only included patients who underwent a first-attempt mitral valve replacement (MVR) using a CM or an SJM mechanical valve. The exclusion criteria included (1) patients who refused to join this study or to sign the consent form; (2) a follow-up period of insufficient length; and (3) patients with other valve diseases, coronary heart disease, or macrovascular disease requiring concurrent surgical management. Follow-up assessments included clinical examination, ECG, chest X-ray, and transthoracic echocardiography (TTE) and were conducted in the 1st, 3rd, 6th and 12th months after the operation.

We used the Chinese version of the short-form health survey (SF-36) to assess the QOL of those patients [5–7]. The SF-36 has already been indicated to be reliable and valid in previous studies and is widely used in China. This questionnaire consists of 36 items with 8 scales (physical role, physical functioning, vitality, bodily pain, emotional role, social functioning, mental health, and general health). A higher score suggests a higher QOL. Patients completed the questionnaire 1 day before the operation in the outpatient department during the follow-up period. Some volunteers helped patients complete the survey through civilian interpretation or translation into local pronunciation but did not interfere with the patient’s choice. Two other independent researchers collected and analyzed the data.

### Statistical analysis

Continuous variables are expressed as x ± s, t-test or analysis of variance was applied for continuous variables, and the χ2 or Fisher’s test was applied for categorical variables. We defined a *P* value< 0.05 as indicative of statistical significance. We used Spearman’s correlation coefficient for ranked data to analyze the correlation between the degree of disturbance due to valve noise and the SF-36 score. We used logistic regression to analyze the following factors: BMI (< 24 vs ≥24), valve type (CM vs SJM), valve size, age (< 60 years vs ≥60 years), and sex (male vs female) in terms of the degree of disturbance due to valve noise. We defined “not disturbing, somewhat disturbing” as 0 and “quite disturbing, very disturbing” as 1 in logistic regression analysis.

## Results

No significant difference was observed in the mean age of the two groups (60.71 years in the CM group and 61.33 years in the SJM group). The current median New York Heart Association (NYHA) status was grade II in both the CM and SJM groups. The clinical characteristics are shown in Table 1.

**Table 1:** Characteristics of enrolled patients

Characteristics	CM group	SJM group	*P* value
Number	52	51	
Age (years)	61.33 ± 6.74	60.71 ± 5.73	0.62
Male(%)	51.92%	50.98%	0.76
Body Mass Index (BMI)	22.99 ± 2.46	22.59 ± 2.25	0.39
Diameter of implanted valve (mm)	27.63 ± 1.28	27.89 ± 1.35	0.32
Current NYHA (median)	II	II	

**Fig. 1:**
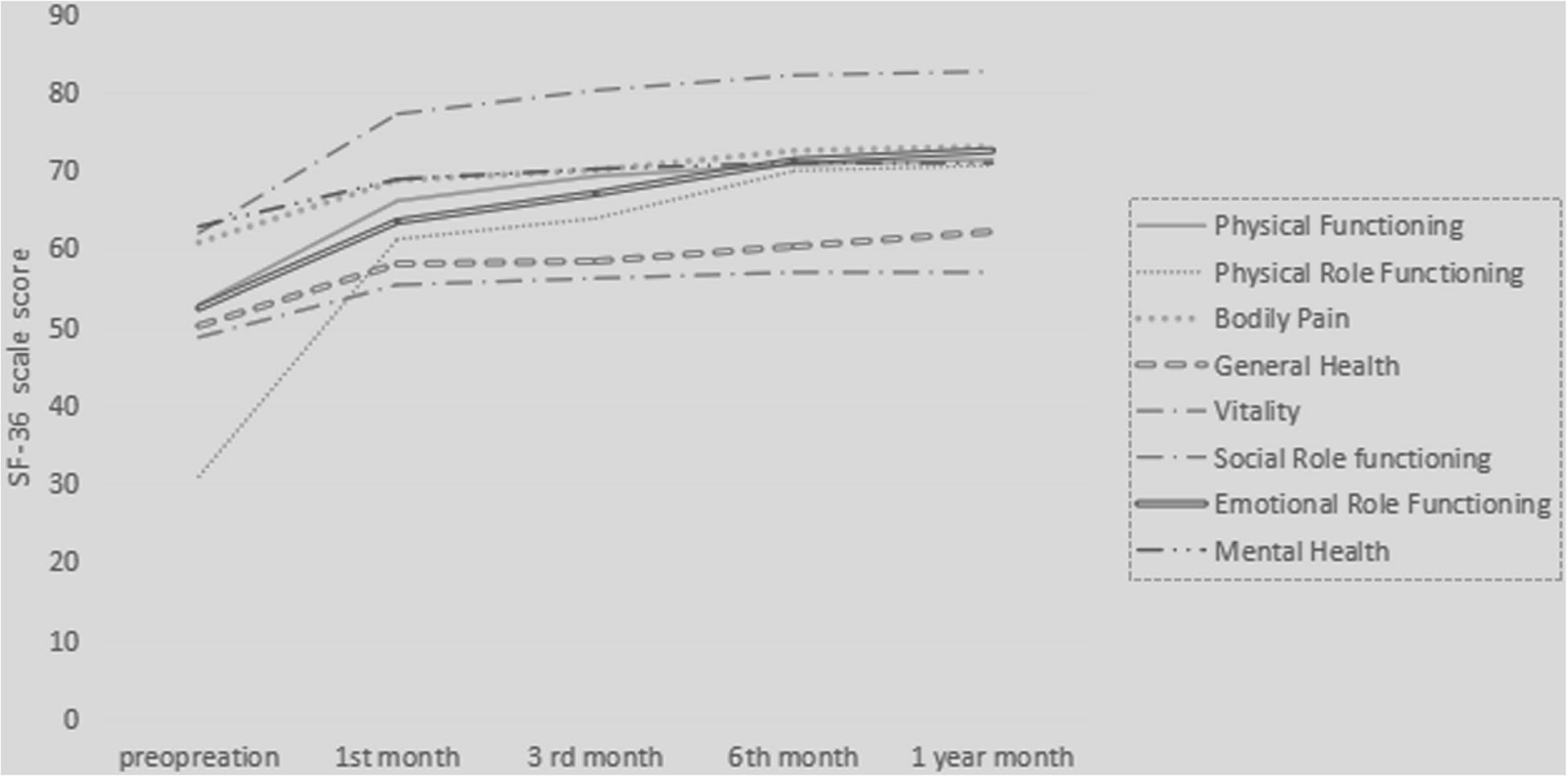
The changes in these eight scales of SF-36 during the follow-up

Table 2 lists the comparison of SF-36 scores from preoperation to 1 year after the operation and the comparison of SF-36 scores between the CM group and SJM group 1 year after the operation. All eight scales of the SF-36 demonstrated significant improvements, which suggests that mechanical MVR can improve patients’ QOL. The changes in these eight scales over time are shown in Fig. 1. The line chart shows an increasing trend of SF-36 scores over time during the follow-up period. The eight scales were similar in the CM and SJM groups (*P* > 0.05) 1 year after the operation.

**Table 2:** SF-36 scale results in preoperation and 1 year after operation (*n* = 103)

Scale	Preoperation	1 year after operation			P^a^	P^b^
		Total	CM group	SJM group		
Physical Functioning	52.86 ± 9.53	71.36 ± 5.76	71.35 ± 5.87	71.37 ± 5.75	< 0.01	0.98
Physical Role Functioning	30.87 ± 16.48	70.63 ± 16.13	70.67 ± 16.21	70.59 ± 16.36	< 0.01	0.97
Bodily Pain	60.75 ± 12.23	73.03 ± 8.45	72.93 ± 62	73.14 ± 8.44	< 0.01	0.90
General Health	50.15 ± 7.58	62.14 ± 6.22	61.92 ± 6.58	62.35 ± 5.95	< 0.01	0.73
Vitality	48.69 ± 7.37	57.09 ± 6.01	56.92 ± 6.50	57.25 ± 5.60	< 0.01	0.78
Social Role Functioning	61.99 ± 18.33	82.57 ± 13.76	82.21 ± 15.13	82.94 ± 12.50	< 0.01	0.79
Emotional Role Functioning	52.43 ± 27.73	72.51 ± 19.94	71.81 ± 20.22	73.22 ± 20.02	< 0.01	0.72
Mental Health	62.80 ± 4.66	71.24 ± 7.14	71.08 ± 7.15	71.41 ± 7.27	< 0.01	0.81

P^a^: comparions of SF-36 scale results between preoperation and 1 year after operation. P^b:^ comparions of SF-36 scale results between CM group and SJM group in 1 year after operation

The coefficient of rank correlation between the SF-36 scores and the degree of disturbance due to valve noise is shown in Table 3. Patients who experienced a greater disturbance due to valve noise demonstrated lower SF-36 scores on each scale. We observed that general health, vitality, and mental health were strongly correlated with the degree of disturbance due to valve noise. Moreover, the other five scales (including physical functioning, physical role functioning, bodily pain, social role functioning, and emotional role functioning) were mildly correlated with the degree of disturbance due to valve noise.

**Table 3:** Coefficient of rank correlation between SF-36 scale results and degree of disturbed by valve noise

Scale	Coefficient of rank correlation	*P* value
Physical Functioning	−0.78	0.00
Physical Role Functioning	−0.78	0.00
Bodily Pain	−0.76	0.00
General Health	−0.83	0.00
Vitality	−0.87	0.00
Social Role functioning	−0.64	0.00
Emotional Role Functioning	−0.67	0.00
Mental Health	−0.82	0.00

**Fig. 2:**
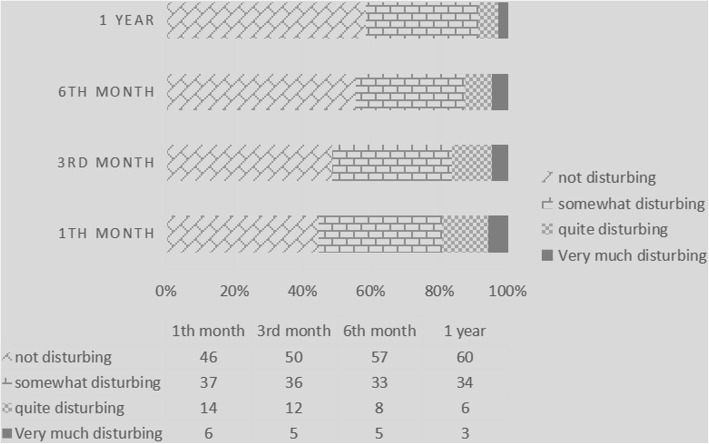
Degree changes of patients disturbed by valve noise during the follow-up

The changes in the degree of disturbance due to valve noise over time in patients are shown in Fig. 2. We categorized the degree of disturbance into 4 levels: not disturbing, somewhat disturbing, quite disturbing, very disturbing. There was no significant difference in these four categories of degree over time (*P* = 0.58). However, the number of patients who experienced disturbances due to valve noise decreased over time. Only 8.74% (*n* = 9) of patients complained of valve noise 1 year after the operation compared to 19.42% (*n* = 20) in the first month after the operation.

The effects of BMI (< 24 vs ≥24), valve type (CM vs SJM), valve size (25 mm vs 27 mm vs 29 mm vs 31 mm), age (< 60 years vs ≥60 years**)**, and sex (male vs female) on the degree of disturbance due to valve noise according to logistic regression analysis are shown in Table 4. We found that female sex and age <  60 years old were high risk factors for experiencing disturbances due to valve noise. Valve type (CM vs SJM), BMI, and valve size showed no significant difference regarding the degree of disturbance due to valve noise.

**Table 4:** Effect of BMI, valve type, valve size, age gender on the degree of disturbed by valve noise by logistic regression analysis

Factor	Comparisons	OR(95% CI)	*P* value
Valve type	CM vs SJM	0.79 (0.30~2.12)	0.64
Valve size (mm)	25 vs 27 vs 29 vs 31	1.06 (0.72~1.55)	0.78
Gender	Male vs Female	4.43 (1.64~11.97)	0.03
BMI	< 24.0 vs ≥24.0	1.25 (0.47~3.33)	0.65
Age (years)	< 60 vs ≥ 60	24.92 (5.04~123.33)	0.00

## Discussion

Mechanical valve replacement has already been proven to be a reasonably safe and effective procedure for valvular heart disease. Although the mortality, morbidity, and recurrence rates of diseases associated with mechanical valve replacement have been assessed in previous studies, the effect of mechanical valves on the quality of life of patients has rarely been studied, especially in Chinese populations. QOL may be affected by the following factors: mechanical valve noise, patients’ mental state, patients’ knowledge of anticoagulation and mechanical valve-related complications. To our knowledge, hemodynamics, antithrombogenicity, and durability of the CM and SJM mechanical valves have already been proven to be had already been proved to be reliable.

Mechanical valves generate a clicking sound that is often audible to patients and even patients’ relatives [7, 8]. However, cardiac surgeons may underestimate the impact of this continuous valve noise on patients’ QOL, unlike life-threatening complications, including anticoagulation and thromboembolic events. Moritz A reported that more than half of patients who underwent mechanical valve replacement could hear the “clicking” sound. The clicking sounds of mechanical valves are considered a source of disturbance and can result in annoyance, sleeping disorders, concentration disturbances and social embarrassment in some cases [9]. A 55-year-old patient experienced severe difficulty in terms of the “clicking” noise made by the mechanical valve. Thus, he underwent a second sternotomy and a second valve replacement with a bioprosthesis valve 4 months after the first mechanical valve replacement. The authors emphasized that the potential risk of valve noise on patients’ QOL should be taken into consideration when choosing an artificial valve [10]. D. Limb also reported that patients and patients’ partners were annoyed by continuous valve noises during sleep, which may lead to reduced concentration and may be detrimental to social relationships [11]. Thus, we focused on the degree of disturbance due to valve noise and QOL in patients who underwent mechanical valve replacement. We also aimed to compare the CM and SJM mechanical valves with respect to the degree of disturbance due to valve noise and the impact on QOL in patients. Golczyk K and his colleagues compared the sound pressure of ATS, SJM and Sorin mechanical valves regarding sound pressure and the degree of disturbance. They found that the sound pressure was difference in each of the above-described three valves. Further, a lower sound pressure demonstrated a better subjective sensation for the patients [12]. Nishi K and his colleagues used a self-administered questionnaire to evaluate patients’ assessments of valve sounds and the SF-36 to measure QOL. The authors found that a long valve sound reduced patients’ QOL as measured by the SF-36 [13]. With the use of a document search, we did not find any studies focusing on the comparison of QOL in patients undergoing mitral valve replacement with CM and SJM, especially in Chinese populations. Thus, we hypothesized that the CM valve and SJM valve had a similar impact on health-related QOL in patients who underwent mitral valve replacement.

All patients in this study completed the SF-36 and provided information about disturbances due to valve-related noise. We found that SF-36 scores in all eight scales increased over time, and this result suggested that patients’ QOL improved over time after mechanical valve replacement, which may be related to excellent postoperative hemodynamics. During the same period, the number of patients experiencing disturbances due to valve noise decreased over time. Only 8.74% (*n* = 9) of patients complained of valve noise 1 year after the operation compared to 19.42% (*n* = 20) in the 1st month after the operation, which suggests that patients may gradually adapt to the effects of valve noise. This change was consistent with the results of other previous reports. Koertke H reported that the percentage of patients who were not or were only somewhat annoyed by valvular noise increased from 90.2 to 94.6% in the 2-year follow-up [2]. Sezai A and his colleagues reported that the percentage of patients who underwent mitral valve replacement and experienced disturbances due to valve noise decreased from 43.5% 1 month after the operation to 13.0% 1 year after the operation [14].

The coefficient of rank correlation between the SF-36 scores and the degree of disturbance due to valve noise showed that patients who experienced disturbances due to valve noise had smaller SF-36 scores on all eight scales. Overall health, vitality and mental health were strongly correlated with the degree of disturbance due to valve noise. The other five scales were mildly correlated with the degree of disturbance due to valve noise. However, it is still unclear whether poor QOL is the result of valve noise or is the cause of valve noise.

Blome-Eberwein and his colleagues reported that complaints about valve sounds had no significant relationship with age, sex, valve type, valve position, or heart rhythm [15]. Laurens and his colleagues reported that complaints about valve sounds were not related to sex, height, weight, or body surface area and that younger patients with mitral valve replacement complained more than older patients with aortic valve replacement [16]. Koertke H and his colleagues reported that valve type, size or site did not have a significant relationship with valve noise perception. They concluded that age less than 60 years and female sex were significantly correlated with valve noise complaints [2]. In the present study, we found that female sex and age <  60 years were high risk factors for experiencing disturbances due to valve noise. We contribute this change in the response to valve noise to physically impaired hearing abilities in those older than 60 years. The clicking sounds of valves are associated with high frequencies, and elderly patients have difficulties hearing these high frequencies [8].

The closure of mechanical heart valves generates an impulse that is transmitted to the patient’s inner ear via two routes: as acoustically transmitted sound waves and as vibrations transmitted through bones and vessels. The difference between males and females may be due to the fact that there is a different resonance reservoir in the thorax, which may be why female patients are more susceptible to interference than male patients. Valve type (CM vs SJM), BMI, and valve size showed no significant differences regarding the degree of disturbance due to valve noise. D. Limb and his colleagues reported that only a few patients had received information about the “clicking” noise [11]. Thus, when this problem occurred, most patients were poorly prepared. It is essential to inform patients who undergo valve replacement to prepare for potential valve noise. If possible, a meeting between patients and someone who has already undergone mechanical valve replacement should be arranged, and the influence of valve noise before undergoing valve replacement should be communicated.

Cardiac surgeons need to inform patients of the potential noise disturbances caused by mechanical valves. Careful preoperative patient teaching, hearing examinations and stimulation of valve noise should be undertaken as routine in the preparation for mechanical valve replacement, especially for patients aged less than 60 years and for female patients [11, 15]. For high-risk patients, a biological valve may be another choice of prosthetic valve [17, 18]. Marc Kottmaier and his colleagues compared QOL and anxiety in younger patients who had undergone biological versus mechanical aortic valve replacement. The authors concluded that valve replacement with a biological prosthesis could be justified with regards to QOL [18].

There are some limitations to this study. First, this study was conducted in a single institution in China. Second, the follow-up period was short. Third, this was a retrospective analysis, not a prospective study, with selective bias. Finally, the sound pressure of the CM and SJM mechanical valves should have been measured to provide more evidence. Therefore, a multicenter study with a larger sample size and longer follow-up will be implemented in future studies.

## Conclusion

The QOL evaluated by the SF-36 improved over time in Chinese patients who underwent mechanical mitral valve replacement. Age less than 60 years and female sex were high risk factors for experiencing disturbances due to valve noise. The CM and SJM mechanical valves were similar in terms of the degree of disturbance and impact on QOL. Further studies with larger sample sizes and longer follow-ups will be necessary to prove the validity of this conclusion.

## Data Availability

Data sharing not applicable to this article as no data sets were generated or analyzed during the current study.

## Abbreviations

BMI: Body mass index
MVR: Mitral valve replacement
NYHA: New York Heart Association
QOL: Quality of life
TTE: Transthoracic echocardiography

## References

[1] Yoganathan AP, He Z, Casey Jones S (2004). Fluid mechanics of heart valves. Annu Rev Biomed Eng.

[2] Koertke H, Hoffmann-Koch A, Boethig D, Minami K, Breymann T, El-Arousy M, Seifert D, Koerfer R (2003). Does the noise of mechanical heart valve prostheses affect quality of life as measured by the SF-36 questionnaire?. Eur J Cardiothorac Surg.

[3] Landolt MA, Buechel EV, Latal B (2011). Predictors of parental quality of life after child open heart surgery: a 6-month prospective study. J Pediatr.

[4] Aicher D, Holz A, Feldner S, Köllner V, Schäfers HJ (2011). Quality of life after aortic valve surgery: replacement versus reconstruction. J Thorac Cardiovasc Surg.

[5] Zhang X, Xia R, Wang S, Xue W, Yang J, Sun S, Zhuang G (2018). Relative Contributions of Different Lifestyle Factors to Health-Related Quality of Life in the Elderly. Int J Environ Res Public Health.

[6] Han L, Li Y, Yan W, Xie L, Wang S, Wu Q, Ji X, Zhu B, Ni C (2018). Quality of life and influencing factors of coal miners in Xuzhou, China. J Thorac Dis.

[7] Xiao Y, Wang H, Zhang T, Ren X (2019). Psychosocial predictors of physical activity and health-related quality of life among Shanghai working adults. Health Qual Life Outcomes.

[8] Nygaard H, Johansen P, Riis C, Hasenkam JM, Paulsen PK (1999). Assessment of perceived mechanical heart valve sound level in patients. J Heart Valve Dis..

[9] Moritz A, Steinseifer U, Kobinia G, Neuwirth-Riedl K, Wolters H, Reul H, Wolner E (1992). Closing sounds and related complaints after heart valve replacement with St Jude medical, Duromedics Edwards, Björk-Shiley Monostrut, and Carbomedics prostheses. Br Heart J.

[10] Kerendi F, Guyton RA (2005). Replacement of mechanical mitral valve prosthesis due to patient intolerance of clicking noise: case report. J Heart Valve Dis..

[11] Limb D, Kay P, Murday A (1992). Problems associated with mechanical heart valve sounds. Eur J Cardiothorac Surg.

[12] Golczyk K, Kompis M, Englberger L, Carrel TP, Stalder M (2010). Heart valve sound of various mechanical composite grafts, and the impact on patients' quality of life. J Heart Valve Dis.

[13] Nishi K, Eishi K, Shibata Y, Amano J, Kaneko T, Okabayashi H, Takahara Y, Takanashi S, Tanemoto K, Yamaguchi H, Kawazoe K (2010). Influence of prosthetic heart valve sound on a patient's quality of life. Ann Thorac Cardiovasc Surg.

[14] Sezai A, Shiono M, Orime Y, Hata H, Yagi S, Negishi N, Sezai Y (2000). Evaluation of valve sound and its effect on ATS prosthetic valves in patients’ quality of life. Ann Thorac Surg.

[15] Blome-Eberwein SA, Mrowinski D, Hofmeister J, Hetzer R (1996). Impact of mechanical heart valve prosthesis sound on patients’ quality of life. Ann Thorac Surg.

[16] Laurens RR, Wit HP, Ebels T (1992). Mechanical heart valve prostheses: sound level and related complaints. Eur J Cardiothorac Surg.

[17] Aboud A, Breuer M, Bossert T, Gummert JF (2009). Quality of life after mechanical vs. biological aortic valve replacement. Asian Cardiovasc Thorac Ann.

[18] Kottmaier M, Hettich I, Deutsch MA, Badiu C, Krane M, Lange R, Bleiziffer S (2017). Quality of life and anxiety in younger patients after biological versus mechanical aortic valve replacement. Thorac Cardiovasc Surg.

